# The “Cap and Gown”

**Published:** 1845-06

**Authors:** 


*The "Cap and Gown."*—An unanimous resolution has been adopted by nearly 500 students of University College, in Upper Gower street, London, in favor of the wearing of an "academical costume," which they regard as being "well calculated to be of advantage to the Institution, to promote the dignity of the College, and to improve the character and morals of the pupils."—*Ibid.*

